# Proteome-based identification of apolipoprotein A-IV as an early diagnostic biomarker in liver fibrosis

**DOI:** 10.18632/oncotarget.21627

**Published:** 2017-10-06

**Authors:** Pei-Wen Wang, Yu-Ching Hung, Tung-Ho Wu, Mu-Hong Chen, Chau-Ting Yeh, Tai-Long Pan

**Affiliations:** ^1^ Department of Medical Research, China Medical University Hospital, China Medical University, Taichung, Taiwan; ^2^ Department of Chinese Internal Medicine, Chang Gung Memorial Hospital-Kaohsiung Medical Center, Kaohsiung, Taiwan; ^3^ School of Traditional Chinese Medicine, Chang Gung University, Taoyuan, Taiwan; ^4^ Division of Cardiovascular Surgery, Veterans General Hospital, Kaohsiung, Taiwan; ^5^ Department of Psychiatry, Taipei Veterans General Hospital, Taipei, Taiwan; ^6^ Department of Psychiatry, College of Medicine, National Yang-Ming University, Taipei, Taiwan; ^7^ Liver Research Center, Chang Gung Memorial Hospital, Taoyuan, Taiwan; ^8^ Research Center for Chinese Herbal Medicine and Research Center for Food and Cosmetic Safety, College of Human Ecology, Chang Gung University of Science and Technology, Taoyuan, Taiwan; ^9^ Chinese Herbal Medicine Research Team, Healthy Aging Research Center, Chang Gung University, Taoyuan, Taiwan

**Keywords:** hepatic fibrosis, proteome, dimethylnitrosamine, ApoA4, network analysis

## Abstract

Hepatic fibrosis may ultimately result in organ failure and death, a reality compounded by the fact that most drugs for liver fibrosis appear to be effective only if given as a prophylactic or early treatment. In a dimethylnitrosamine-induced liver fibrotic model, aspartate aminotransferase/alanine aminotransferase levels could not precisely distinguish the differences between the initial stage of liver fibrosis and normal control, whereas histological examination indicated that dimethylnitrosamine treatment for two weeks has resulted in hepatic fibrogenesis. Comprehensive proteomics identified 12 proteins mainly associated with the interleukin 6-stimulated inflammatory pathway. Coordinately, cytokine profiles showed that dimethylnitrosamine administration would stimulate various signaling pathways leading to liver fibrosis. Of note, apolipoprotein A4 in serum samples obtained from patients in the early stage of liver fibrosis were significantly increased compared to the healthy controls (*p*<0.001) while the area under curve was 0.966. Moreover, increased apolipoprotein A4 significantly enhanced transforming growth factor beta 1-induced alpha smooth muscle actin expression. In this regard, overexpression of apolipoprotein A4 in early stage of liver fibrosis might magnify and imply the progression of hepatic fibrosis. These findings suggest that apolipoprotein A4 upregulation may correlate with hepatic fibrosis staging and that apolipoprotein A4 together with current biomarker can increase the sensitivity and specificity for the early detection of liver fibrosis in a high-throughput manner.

## INTRODUCTION

Liver fibrosis is the pathological response to chronic liver disease characterized by excessive deposition of extracellular matrix (ECM) proteins and regenerative nodules, leading to impairment of liver function [1]. The eventual result of uncontrolled liver fibrosis leads to liver failure, portal hypertension, and increased risk of hepatoma [2]. Because the early stage of liver fibrosis is asymptomatic and distinctive clinical signs do not appear until the disease is advanced, the incidence of liver-fibrosis-associated diseases is rising, as are mortality rates.

As the burden of liver problems increases, our therapeutic strategy for the treatment of liver fibrosis becomes more and more challenging due to a lack of convenient, readily available methods for early diagnosis, which is usually achieved by invasive or expensive examinations [3]. In addition, indirect measurements of decreased liver function do not dependably reflect abnormality until hepatic disorder is considerably advanced. Therefore, detecting biological markers that could be accurately and easily used to assess hepatic fibrosis prior to the appearance of more severe injury is critical [4]. In this regard, a serological sample is a feasible tool for searching for early diagnostic biomarkers because blood collection is minimally invasive and highly convenient [5].

Clinical samples are difficult to collect and also pose the challenge of personal variation. It is therefore reasonable to use experimental animal models for liver fibrosis research to perform the complex progression that results in hepatic fibrogenesis. Several triggers induce hepatic fibrosis in animal models [6]. These include bile duct ligation, ethanol feeding, and hepatotoxin reagents including of thioacetamide (TAA), dimethylnitrosamine (DMN), and CCl_4_. Several reports have demonstrated that DMN could rapidly induce liver fibrosis within two weeks through metabolic activation [7]. A DMN-caused hepatic fibrosis model would generate most of the features, including ascites, ECM accumulation, and histopathological changes as observed in human liver fibrosis [8]. This model, which is also stable even after termination of DMN administration, is a reliable tool for studying issues associated with hepatic fibrosis.

During liver fibrosis, great amounts of proteins will be altered in quantity and quality. Changes in levels of these proteins may reflect the onset and progression of the disease states. Proteome tools that integrated two-dimensional electrophoresis and mass spectrometry allowed high-throughput analyses to discover novel diagnostic biomarkers by revealing differential protein expression profile [9]. Furthermore, we comprehensively represented protein profiles associated with “signature networks” [10], and we generated global cellular mechanisms involved in protein responses to DMN-induced hepatic fibrogenesis.

Taken together, clinical biochemistry still needs to be improved with respect to early marker detection in liver fibrosis. In the current study, we aimed to identify novel plasma biomarkers of liver fibrosis using a proteome-profiling method. With this integrated approach, the valuable role of the ApoA4 level was determined in predicting early hepatic fibrosis.

## RESULTS

### Determination of plasma ALT and AST as well as histopathological changes in rat liver tissues under DMN treatment

**Figure 1:**
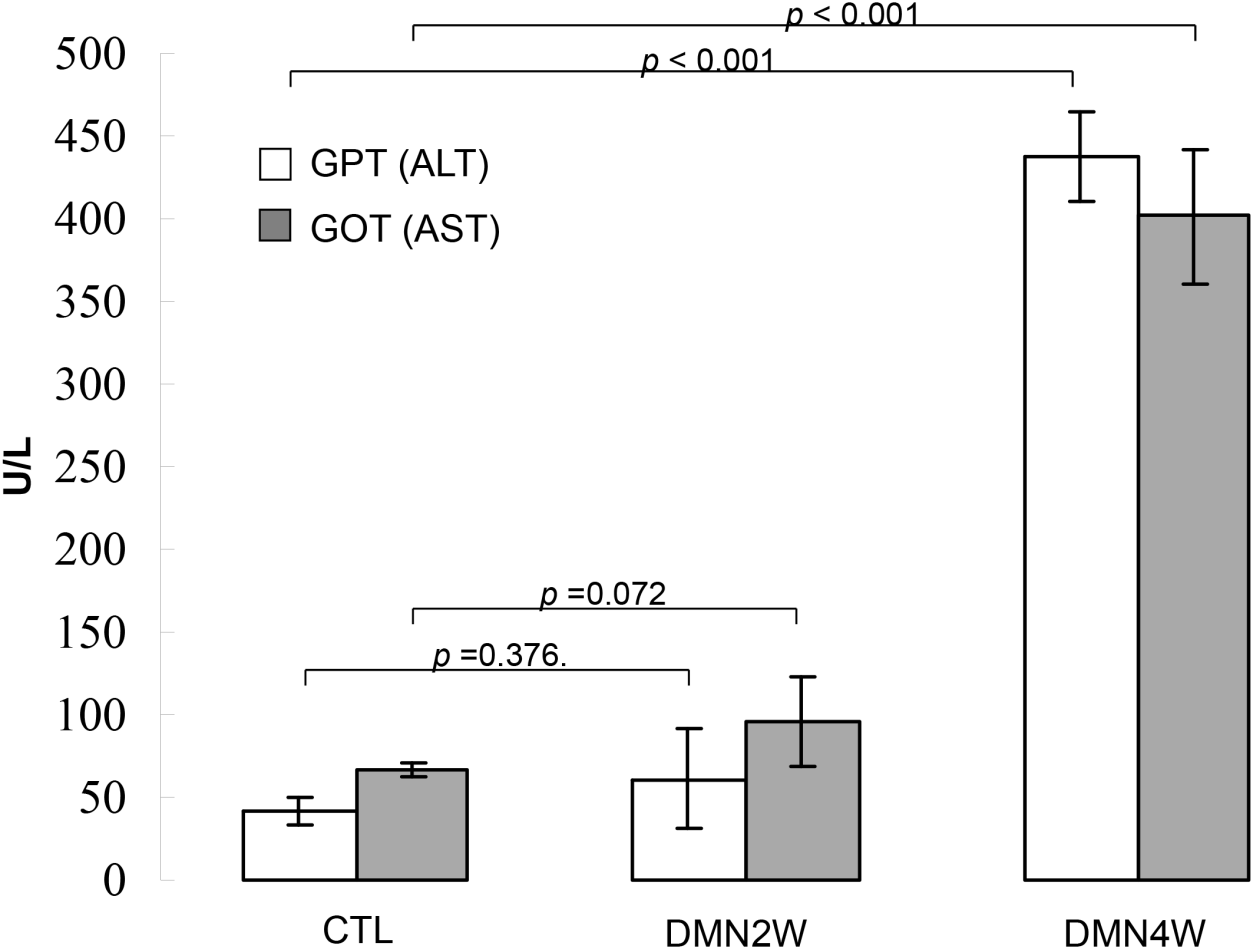
Validation of AST and ALT levels in plasma samples obtained from the rats treated with DMN for two weeks (DMN2W), four weeks (DMN4W) and control (CTL), respectively Data are means ± SD of the independent experiments.

**Figure 2:**
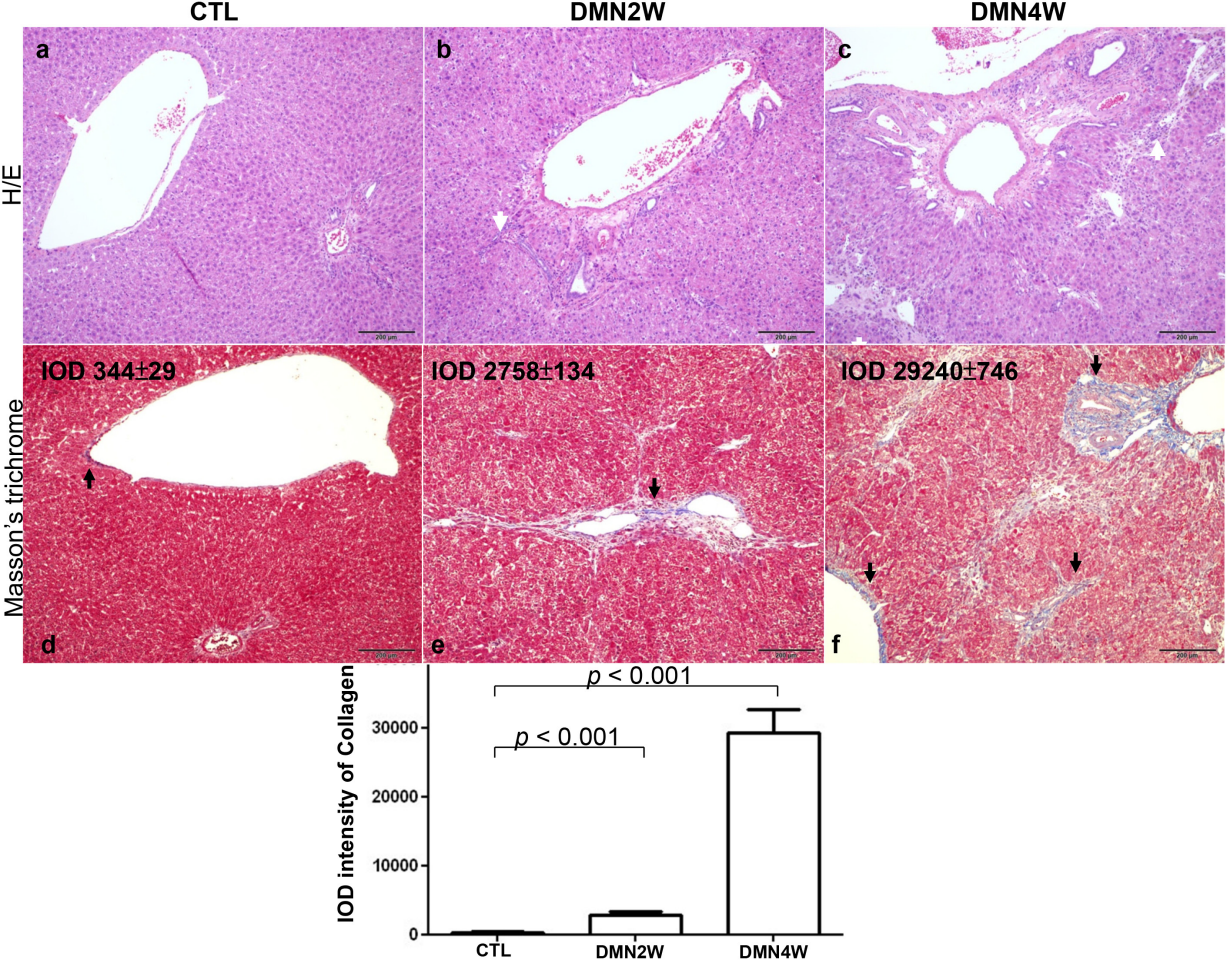
Upper panels: histologic examination of rat liver by H/E staining The white arrows indicated necrosis of hepatocytes and infiltrated lymphocytes. Lower panels: Masson’s trichrome staining of rat liver tissues. The images demonstrate the representative image of control and DMN-treated samples. The black arrows showed accumulation of collagen around portal tracts as blue images. (magnification ×200). The protein intensity was quantified using Image Pro-Plus 4.5 computer program. IOD: integrated optical density.

Plasma was collected from different groups for biochemical examination including AST and ALT as depicted in Figure 1. Significantly increased values of AST and ALT (*p*< 0.001) were shown in subjects treated with DMN for four weeks compared with samples applied with DMN for two weeks as well as the control samples. Conversely, both AST and ALT in plasma distinguished poorly between the samples applied with DMN for two weeks and the control group. Nevertheless, administration of DMN for two weeks resulted in necrosis of hepatocytes, inflammatory infiltration (Figure 2b), and early liver fibrogenesis (Figure 2e). As expected, four-week exposure to DMN led to severe hepatic damage, which manifested as massive necrosis of hepatocytes (Figure 2c) and remarkable fibrosis, whereas a great amount of collagen was accumulated (Figure 2f) with the control sample showing intact lobular architecture (Figure 2a & 2d). These results implied that traditional biochemical parameters such as AST and ALT might not be effective indicators for detecting the onset of liver fibrogenesis.

### Differential display of plasma proteins between samples treated with DMN for two weeks and the control

**Figure 3:**
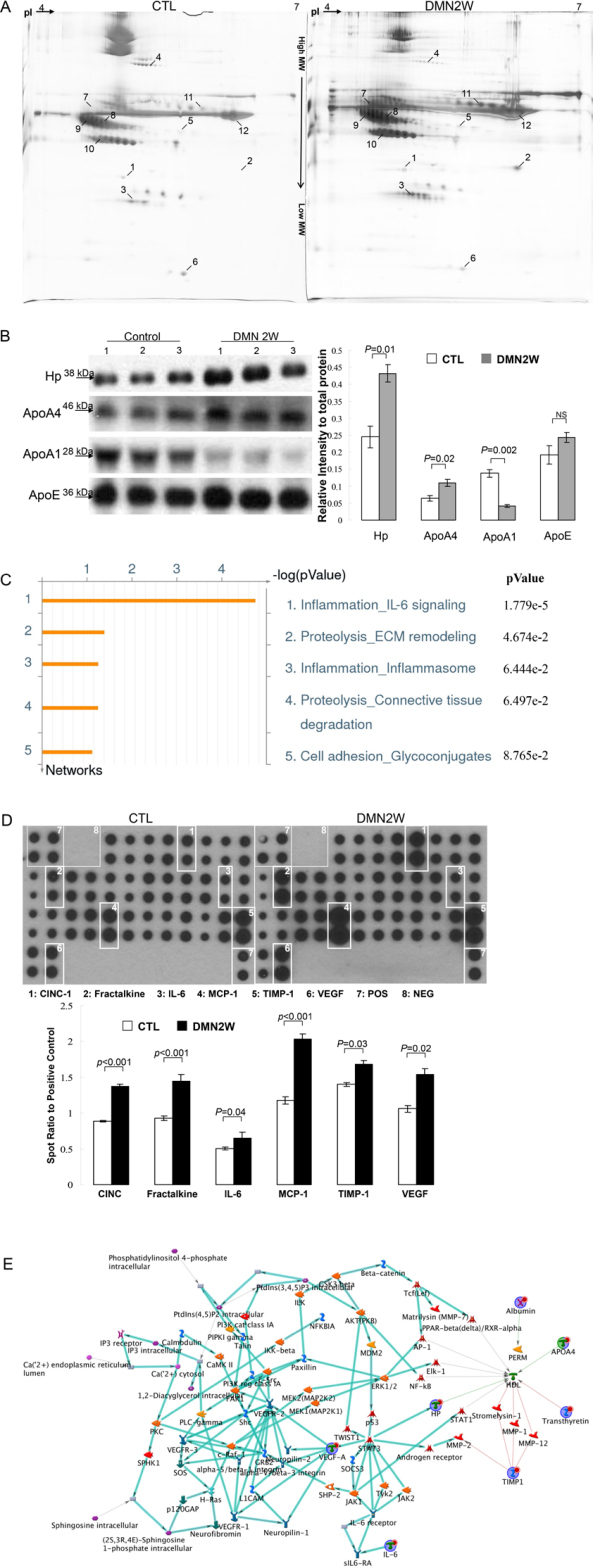
**(A)** Comparison of selected protein spots between control (CTL) and samples exposed to DMN for two weeks (DMN2W). The protein lysate was focused on a pH 4∼7 linear IPG strip and then separated on a 10% polyacrylamide gel. The identified proteins are labeled with Arabic numerals. **(B)** Western blotting was used to determine the expressions of protein targets. The quantitative results were demonstrated as bar charts. **(C)** Top-ranked pathways predicted by the GeneGo MetaCore™ software. Pathways were ranked according to *p* values, and bars represent the inverse log of the *p* value. **(D)** Levels of cytokine/chemokine from the control and DMN2W groups were assessed by protein array. Square numbering: 1: CINC-1; 2: Fractalkine; 3: IL-6; 4: MCP-1; 5: TIMP-1; 6: VEGF; 7: Positive control; 8: Negative control. Lower panel showed the intensity of the chemiluminescent signals for each spot was quantificated by GeneTools software. Expression levels were normalized with respect to positive controls on the array membrane. The quantitative results indicating the different values compared with the control samples were demonstrated as a bar chart. **(E)** Nodes represent proteins and lines between the nodes indicate direct protein–protein interactions. The various proteins on this map are indicated by different symbols representing the functional class of the proteins. The related biologic processes in this network are involved in lipid metabolism and the NF-κB mediated inflammation.

To further characterize the potent biomarkers for early diagnosis of liver fibrosis, functional proteomic strategies provided a powerful route to dissect the global changes of plasma proteins between samples applied with DMN for two weeks and the control. Figure 3A demonstrates the representative gel images of the experimental samples visualized by silver staining. Approximately 756 protein spots appeared in the 2-DE maps, and a computer-assisted analysis of the respective protein spots revealed 12 protein targets indicated by Arabic numerals with significant and meaningful changes (>1.5-fold change). The typical 2-DE maps were presented and the results in triplicate were reproducible. MS/MS analyses unambiguously identified all 12 protein targets. A typical example is shown in Supplementary Figure 2 in which ApoA4, representing 80% sequence coverage, is characterized. A similar trend in the induced protein expression levels, as determined by Western blotting, was shown in the results as they appeared in 2-DE analysis. As presented in Figure 3B, an equal amount of albumin indicated that the loading protein volume for both groups was the same. Table 1 summarizes the integrative results of the spectrometric analyses and protein functions.

**Table 1:** Lists of differently expressed proteins in DMN model

Spot no.	Protein name	Acession no.	Mw (kDa)	pI	Score (coverage)	MS/MS fragment (ions score)^a^	Fold difference^b^	*p*-value^c^	Biological Function
1	Apo A4	P02651	44.43	5.12	452 (80%)	^156^QLTPYIQR^163^ (32)	+6.3±0.2	0.01	May have a role in chylomicrons and VLDL secretion and catabolism. Required for efficient activation of lipoprotein lipase by ApoC-II; potent activator of LCAT.
						^295^QLDQQVEVFR^304^ (64)			
						^295^QLDQQVEVFRR^305^ (25)			
2	TTHY	P02767	15.82	5.77	164 (67%)	^101^ALGISPFHEYAEVVFTANDSGHR^123^ (83)	+5.6±0.6	0.01	Thyroid hormone-binding protein.
3	HPT	P06866	39.05	6.10	241 (40%)	^219^MGYVSGWGR^227^ (51)	+2.4±0.4	0.03	Acts as an antioxidant, has antibacterial activity and plays a role in modulating many aspects of the acute phase response.
						^321^SCAVAEYGVYVR^332^ (64)			
4	A1I3	P14046	165.04	5.70	161 (27%)	^215^EEHSFTVMEFVLPR^228^ (20)	-4.3±0.2	0.01	Protease inhibitor with a wide spectrum of protein targets, which attaches through its thioester function.
						^587^VTASPQSLCGLR^598^ (28)			
5	VTDB	P04276	55.11	5.65	336 (71%)	^42^SLSLILYSR^50^ (40)	+1.8±0.5	0.01	In plasma, it carries the vitamin D sterols and prevents polymerization of actin by binding its monomers. DBP associates with membrane-bound immunoglobulin on the surface of B-lymphocytes and with IgG Fc receptor on the membranes of T-lymphocytes.
						^51^KFPSSTFEQVSQLVK^65^ (103)			
						^353^RTQVPEVFLSK^363^ (40)			
6	Apo A1	P04639	30.10	5.52	317 (70%)	^64^QLNLNLLDNWDTLGSTVGR^82^ (34)	-2.4±0.3	0.02	Participates in the reverse transport of cholesterol from tissues to the liver for excretion by promoting cholesterol efflux from tissues and by acting as a cofactor for the lecithin cholesterol acyltransferase (LCAT).
						^131^WNEEVEAYR^139^ (90)			
						^182^FGLYSDQMR^190^ (45)			
7	KNT1	P01048	48.83	6.08	282 (43%)	^118^FSVATQICNITPGK^131^ (73)	+3.5±0.8	0.04	Kininogens are plasma glycoproteins with a number of functions: (1) as precursor of the active peptide bradykinin they effect smooth muscle contraction, induction of hypotension and increase of vascular permeability. (2) They play a role in blood coagulation by helping to position optimally prekallikrein and factor XI next to factor XII. (3) They are inhibitor of thiol proteases.
						^44^YNAELESGNQFVLYR^58^ (112)			
8	SPA3K	P05545	46.76	5.31	312 (54%)	^188^IAELFSELDER^198^ (58)	+2.9±0.1	0.01	Binds to and inhibits kallikreins. Inhibits trypsin but not chymotrypsin or elastase.
						^312^FSISTDYNLEEVLPELGIR^330^ (22)			
						^332^IFSQQADLSR^340^ (61)			
9	SPA3L	P05544	46.42	5.48	239 (66%)	^188^IAELFSDLEER^198^ (45)	+1.5±0.2	0.01	By growth hormone. Reduced during acute inflammation
10	SPA3N	P09006	46.79	5.33	328 (66%)	^116^GFGHLLQR^123^ (29)	+1.6±0.4	0.03	By acute inflammation
						^157^ALYQAEAFTADFQQSR^172^ (69)			
11	HEMO	P20059	52.06	7.58	228 (49%)	^208^FNPVTGEVPPR^218^ (34)	+2.7±0.1	0.02	Binds heme and transports it to the liver for breakdown and iron recovery, after which the free hemopexin returns to the circulation.
						^270^GATYAFSGSHYWR^282^ (50)			
12	ALBU	P02770	70.68	6.09	217 (41%)	^348^DVFLGTFLYEYSR^360^ (34)	+1.9±0.3	0.04	Its main function is the regulation of the colloidal osmotic pressure of blood.

(a) Protein scores greater than 56 are significant; all the proteins validated should have at least one peptide identified by MS/MS spectra with an individual ion score > 28 indicated identity or extensive homology (*p*< 0.05). The charge state of each peptide is “+1”.(b) Fold difference: increase (+) or decrease (-) in expression in DMN2W *versus* control.(c) *p*-values were generated by analyzing the gel images using Nonlinear Progenesis software. These values are representative of fibrotic plasma compared to control. Differences were considered significant at *p < 0.05.

### Network analysis

MetaCore™ analytical software was used to predict the relationship of targeted proteins revealed by proteomic analysis and the underlying mechanisms associated with the etiology of hepatic fibrogenesis. As demonstrated in Figure 3C, protein–protein interaction networks indicated that differentially expressed proteins resulting from DMN treatment were primarily involved in the following statistically significant networks: inflammation IL-6 signaling (*p*= 1.779×10^−5^), proteolysis ECM remodeling (*p*= 4.674×10^−2^), inflammation inflammsome (*p*= 6.444×10^−2^), proteolysis connective tissue degradation (*p* = 6.497×10^−2^) and cell adhesion glycoconjugate (*p*= 8.765×10^−2^). According to the network analysis, the IL-6 signaling pathway seemed to be involved in liver fibrosis caused by DMN. A high level of saturation of this pathway with topologically significant nodes was in clear agreement with the current views on the effect of DMN for hepatic fibrogenesis.

### Characterize the interactive map by integrating the cytokines and protein profiles

The aforementioned results indicated that DMN application for two weeks might induce the IL-6 cascade involved in liver fibrosis. Next, we used the cytokine array to investigate the global changes of cytokines and growth factors that mediated hepatic fibrogenesis. As demonstrated in Figure 3D, low levels of cytokines and chemokines were shown in the control samples whereas CINC-1, fractalkine, IL-6, MCP-1, TIMP-1 and VEGF significantly increased in the DMN2W subjects. As expected, the results implied that the increased cytokines and chemokines that induced inflammation were responsible for the development of liver fibrosis induced by DMN application. The shortest-path algorithm was applied to generate the map interactions among the proteins revealed by proteome and cytokine profile analysis. According to this network, lipid metabolism is tightly associated with the inflammation, as well as regulation of specific transcriptional factors such as NF-κB, AP-1 as well as PPAR (Figure 3E).

### Plasma levels of ApoA4 in patients with mild hepatic fibrosis

**Figure 4:**
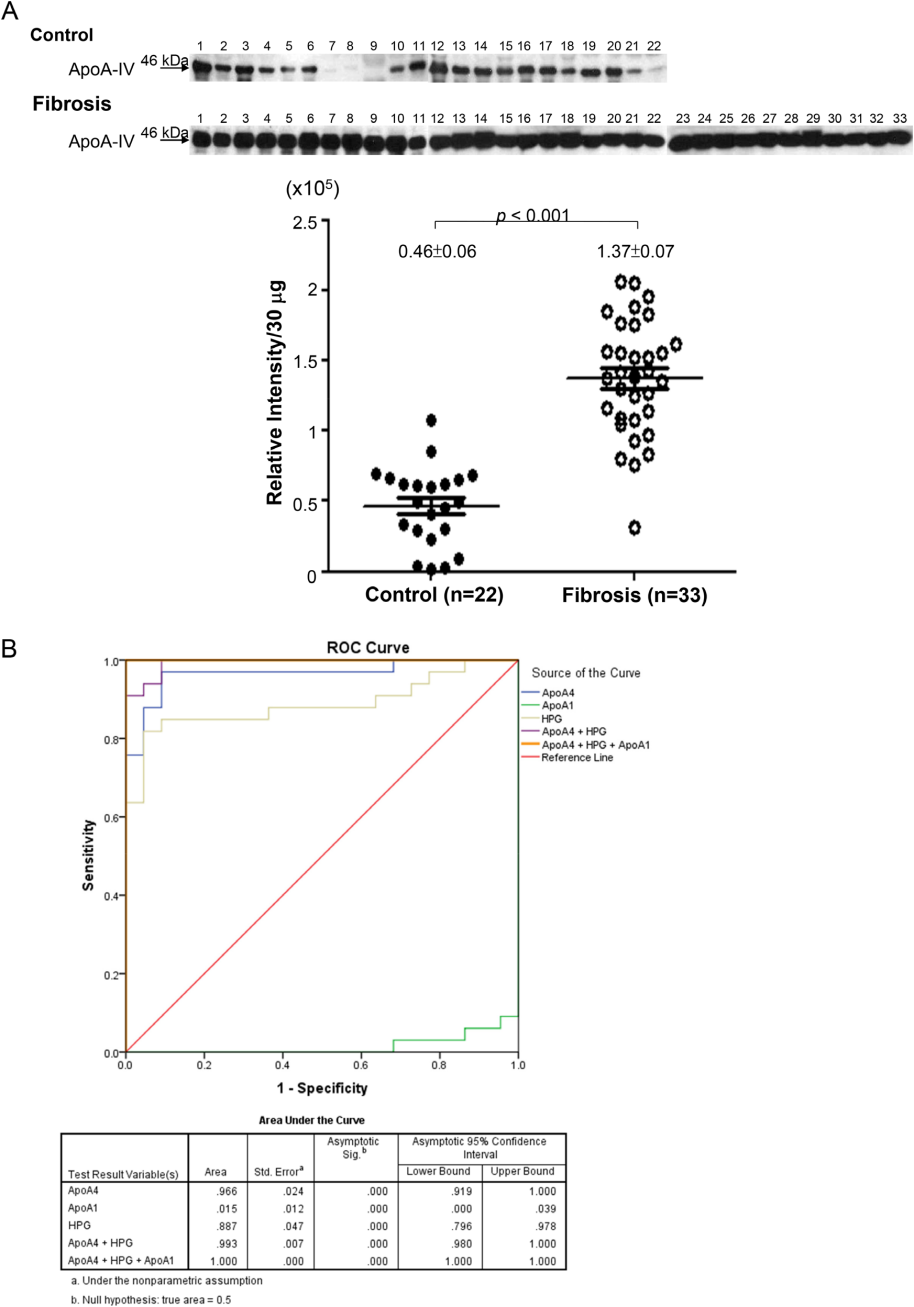
**(A)** The quantitative results indicating the different levels of ApoA4 in clinical plasma specimens between healthy control (n=22) and patients with mild hepatic fibrosis (n=33). **(B)** ROC curve analysis showed that ApoA4 (area under curve: 0.966) and haptoglobin (area under curve: 0.887), but not ApoA1 (area under curve: 0.015), were the good plasma markers for the early identification of liver fibrosis.

Of note, ApoA4 protein was significantly elevated in the plasma samples of rats under DMN treatment for two weeks compared with the normal control. Moreover, pathway analysis also suggested that ApoA4 would be associated with PPAR-caused hepatic fibrogenesis. Meanwhile, some evidence showed that ApoA4 protein expression could be stimulated by hepatic steatosis. Taken together, we evaluated the levels of ApoA4 in the clinical plasma specimens which were obtained from healthy controls and patients with mild hepatic fibrosis. As expected, the levels of ApoA4 in the patients’ plasma was significantly higher than that in the control group (*p*<0.001) as shown in Figure 4A, suggesting that plasma ApoA4 levels may be a potential marker in early diagnosis of hepatic fibrosis.

### ApoA4 improves the score of panel markers in early detection of liver fibrogenesis

ApoA1 and haptoglobin have been applied in the current test system for liver disease. Here, we preliminarily validated the relationship between plasma ApoA1, ApoA4, as well as haptoglobin and liver fibrosis to judge which plasma biomarker is better for the early detection of liver fibrosis (Figure 4A). An ROC curve analysis was constructed to further compare the diagnostic capacity of these potent markers in distinguishing early liver fibrosis from the control. We calculated the AUC value and showed that ApoA4 (area under curve: 0.966) and haptoglobin (area under curve: 0.887) might be the good biomarkers for the early identification of liver fibrosis. Sensitivities and specificities of panel markers (ApoA4+HPG+ ApoA1) for early liver fibrosis detection can be greatly improved (Figure 4B).

### Exogenous ApoA4 promotes alpha smooth actin expression under TGF-β1 application

**Figure 5:**
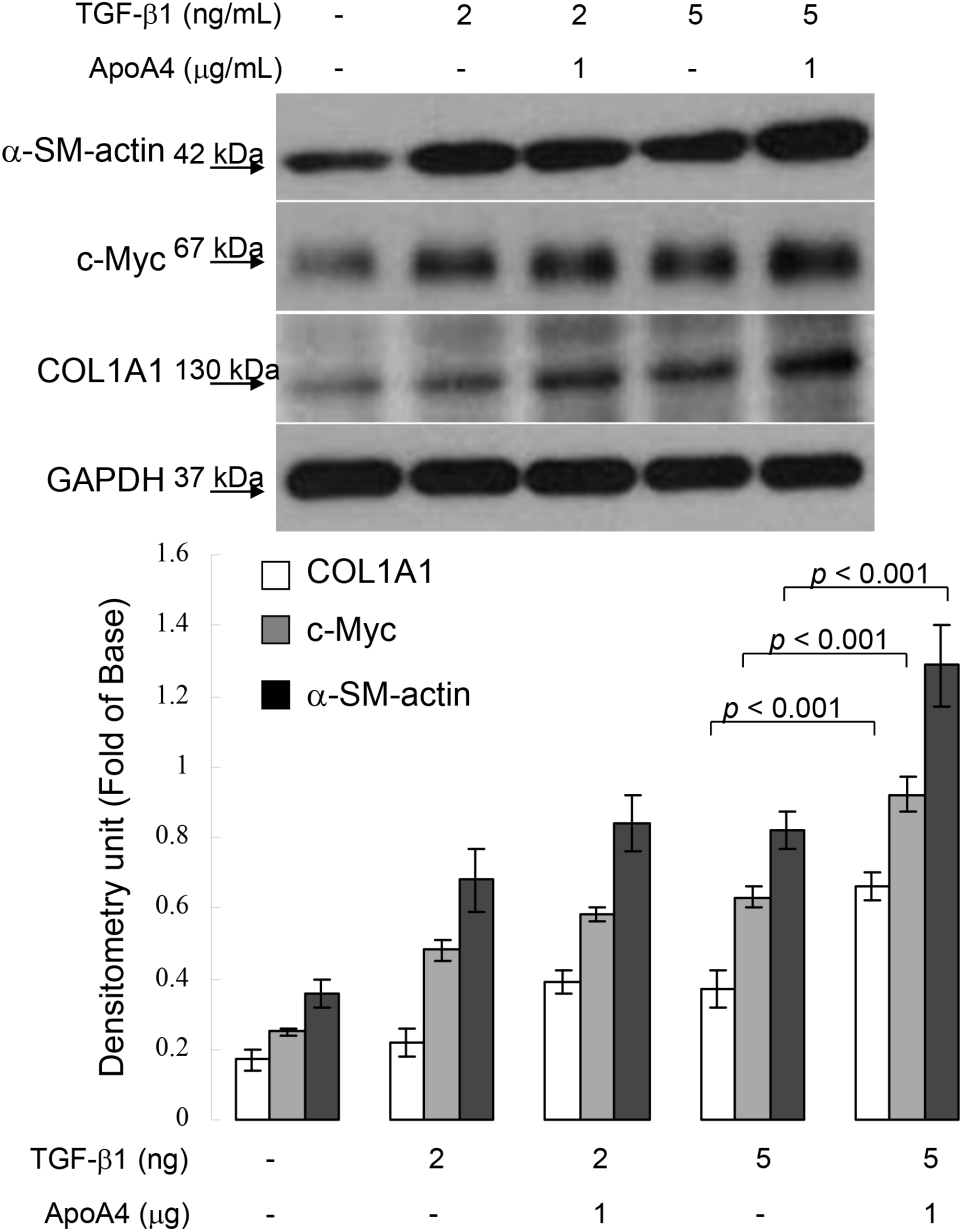
Validation of α-SMA, c-Myc and COL1A1 expression in HSC-T6 cells exposed to TGF-b1 with or without ApoA4 by Western blotting GAPDH was used as an internal control. The quantified results were indicated by the bar chart and represent the mean ± SD of three independent experiments.

To further ascertain the effect of ApoA4 on hepatic fibrogenesis, HSC-T6 cells were exposed to 1 μg/mL ApoA4. As shown in Figure 5, synergistic administration of ApoA4 and TGF-β1 markedly increased in α-SMA and COL1A1 levels with respect to the sample treated with TGF-β1 only. Meanwhile, simultaneous application of ApoA4 and TGF-β1 also stimulated the expression of c-Myc. These results suggest that ApoA4 could induce hepatic fibrosis through activating hepatic stellate cells in the presence of TGF-β1.

## DISCUSSION

Liver fibrosis is a hallmark characteristic of all chronic liver diseases, regardless of the cause, and this healthy impact results in approximately 800,000 deaths per year worldwide. It is more difficult to detect patients with pre-fibrotic disease, because collagen content increases exponentially with each stage. Therefore, the greatest challenge for clinical therapy of liver fibrosis is likely to be realized from the management of early-stage disease rather than from treatment of late-stage disease [11, 12]. Here, the rats treated with DMN for two weeks were considered to be in the early phase of liver fibrosis.

Since fibrosis is the result of liver damage, ALT and AST released from the liver tissue into the circulation should indicate the degree of liver injury and function. Our histological data showed that DMN2W already resulted in mild pathological change in the liver while DMN4W led to advanced hepatic fibrosis. However, there were no significant differences between the control group and the samples treated with DMN for two weeks with respect to the values of ALT and AST. This indicated that ALT and AST might not be a reliable index for early cytopathogenesis in the liver.

Currently, the noninvasive method using the plasma biomarker has offered an effective alternative to liver biopsy, which is usually associated with severe complications for patients [4, 13]. However, available noninvasive assays including the FibroTest and FibroScan could provide the diagnosis for advanced liver fibrosis, they are unlikely to be accurate enough to detect the subtle changes at the earlier stages of the disease [14]. The emerging field of proteomics provides a global view and high expectations for plasma biomarker discovery. In particular, the quantitative proteome method based on changes in protein levels offers insight into the pathology of fibrosis and provides possibilities for novel selective bioassays [15]. A comparison of plasma proteomes demonstrated that DMN treatment for two weeks induced not only the initial stage of liver fibrosis but also subtle alterations of several plasma proteins belonging to numerous physiologic pathways. Twelve protein spots displayed a significant difference in intensity under DMN treatment compared to the control. Of these targeted proteins, HPT and ApoA1 also scored in the FibroTest, indicating that proteome analysis is a feasible tool for diagnosis of liver fibrosis [16].

The targeted proteins used as input for network analysis and the major networks were responsible for IL-6-associated inflammation, extracellular matrix remodeling, and connective tissue degradation. IL-6 is a pleiotropic cytokine in modulating inflammatory responses in liver diseases through activation and phosphorylation of both NF-κB and STAT3 proteins, which in turn stimulate progressive liver fibrosis [17, 18]. Cytokines, chemokines, angiogenic factors and growth factor have been identified as pivotal regulators in fibrogenesis. As indicated in the results of cytokine arrays, samples treated with DMN for two weeks significantly upregulated various factors and molecules that subsequently induced acute inflammation and liver fibrosis. In our study, CINC-1 and MCP-1 markedly stimulated by DMN administration showed the chemotactic activity mediating the accumulation of neutrophils and monocytes, which are involved in acute inflammation [19]. Similarly, the proteome profiles also showed that DMN exposure resulted in an obvious increase in the level of T-kininogen. It belongs to the plasma kallikrein–kinin system and participates in the pathogenesis of inflammatory reactions involved in cellular injury [20]. In addition, levels of TIMP-1 and VEGF, which stimulate the development of hepatic fibrosis, were also promoted under DMN application. Correspondingly, Masson’s trichrome staining results indicated that DMN application might be implicated in ECM turnover and collagen degradation.

Previous reports have further indicated that hepatic lipid metabolism is closely implied in inflammatory, proliferative and apoptotic signaling in the DMN-induced rat model [21]. Interestingly, ApoA1 and ApoA4 which are involved in lipid metabolism showed dramatic changes in protein volume under DMN exposure. It is not surprising that the liver function is associated with changes in apolipoproteins synthesized in the liver [22]. ApoA1 is the major protein component of high-density lipoprotein in the plasma, and a decrease of ApoA1 has been found in the plasma of patients with hepatic cirrhosis [23]. Consistent with our data, a significant reduction of ApoA1 in the clinical plasma and samples obtained from the rats exposed to DMN for two weeks was identified.

On the other hand, ApoA4 was identified in spot 1 that appeared to be significantly upregulated in the early fibrogenesis induced by two-week DMN treatment compared with the control samples. ApoA4, a protein expressed in the mammalian small intestine, appears to have various functions including playing a role as a lipoprotein anti-oxidant, participating in reverse cholesterol transport and being a major factor in the prevention of atherosclerosis [24]. The previous report has also indicated that hepatic steatosis would stimulate hepatic ApoA4 expression, which in turn would reduce the hepatic lipid burden by promoting lipoprotein particle expansion [25]. Excessive hepatic lipid sensitizes hepatocytes to the attack manifested by increased inflammation, induction of oxidative stress, and activation of fibrosis [26]. Moreover, increased ApoA4 could serve as an acute-phase protein to play a role in the host defense needed during inflammation [27]. In this regard, elevated expression of ApoA4 should be associated with metabolic abnormalities of lipids as well as the inflammatory process caused by hepatic impairment.

As mentioned above, ApoA4 may be a novel plasma marker in early diagnosis of hepatic fibrogenesis since this apolipoprotein is more specific to reflect liver pathology. Our results confirmed that ApoA4 was significantly upregulated in plasma samples obtained from subjects with mild liver fibrosis compared to those in the control group. Meanwhile, ApoA4 levels were significant in hepatic fibrosis patients versus the healthy controls (*p*<0.001). Based on AUROC analysis, ApoA4 as an additional marker would increase the sensitivity and specificity in the detection and staging of liver fibrosis. In addition, ApoA4 combined with TGF-β1 could enhance the levels of α-SMA, COL1A1 and c-Myc. Previous study indicated that activated HSCs would enhance more collagen production [28]. In these regards, we suggested that the ApoA4 may reinforce TGF-β1-caused hepatic fibrosis due to the stimulation of HSCs. Determination of the plasma ApoA4 level seems to play a critical role in the early and rapid diagnosis of liver fibrogenesis.

**Figure 6:**
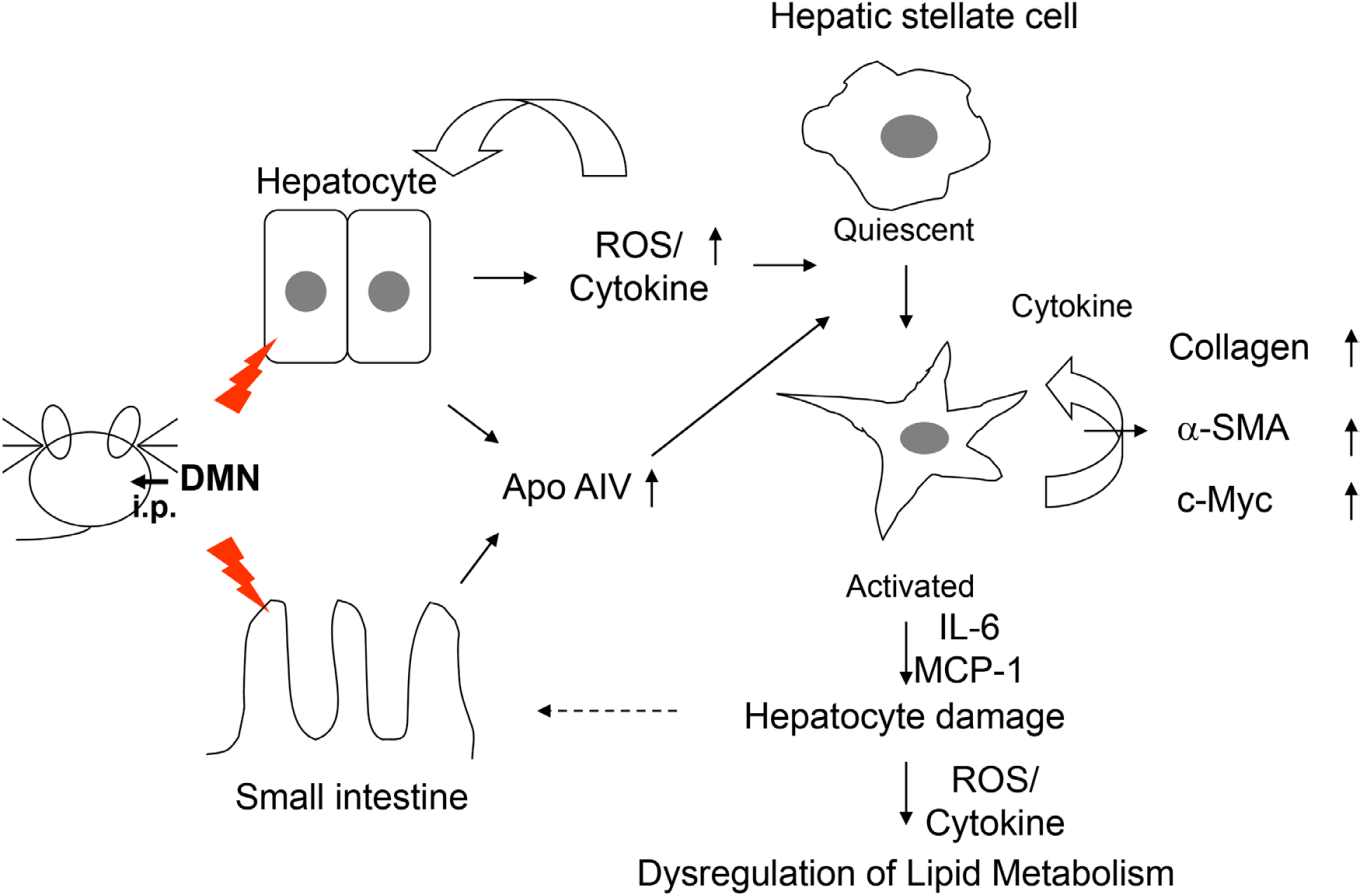
Proposed diagram of ApoA4 mediated activation of hepatic stellate cells in DMN-induced early onset of liver fibrogenesis

In summary, DMN treatment would result in the overproduction of ROS and the release of inflammation-associated cytokines, sequentially turning the quiescent hepatic stellate cell into an activated cell and disrupting retinoid/lipid metabolism. Finally, hepatocyte damage caused by inflammatory cytokine and reactive stellate cells might eventually stimulate the expression of ApoA4, reflecting early pathology in the liver (Figure 6). Our findings provide evidence that ApoA4 might be a key factor in the determination of the inflammatory responses and cell transformation. Therefore, ApoA4, along with additional biochemical parameters, could provide noninvasive assessment of early liver fibrosis, a possibility that warrants further experimental verification.

### Perspective work

According to our findings, metabolism of ApoA4 seems to play a critical role in early stage of liver fibrogenesis. Therefore, we will apply the animal model and intestine cells to further establish the novel contribution of ApoA4 to the liver injury in general as well as to explore molecular mechanisms whereby these cells increase their expression of extracellular matrix components during hepatic fibrosis.

## MATERIALS AND METHODS

### Animals

Wistar rats weighing 200g were purchased from Lasco co. (Taiwan). All rats were maintained in a standard facility and allowed access to food and water. After acclimatizing to laboratory conditions for one week, the rats were randomly divided into individual groups of six each. In the DMN-induced fibrosis group, rats were injected intraperitoneally with DMN (10 mg/kg body weight) for three consecutive days per week [7, 29]. At the end of the two or fourth week, all of the rats were sacrificed. The control group was treated with only vehicles. Their livers were excised and immediately fixed in 10% neutral buffered formaldehyde for pathological studies. The methods were carried out in “accordance” with the approved guidelines and the Committee on Research Involving Animal Subjects of the Chang Gung University, Taiwan, had approved all experimental protocols in this study.

### Plasma biochemical parameters and histology assessment of rat samples

Rat blood samples were collected and immediately centrifuged at 4°C. The rat plasma samples were stored at -80°C before analysis. AST and ALT were measured using a colorimetric analyzer (Dri-Chem 3000, Fuji Photo Film, Tokyo, Japan). A piece of liver tissue fixed by formalin was then embedded in paraffin and sliced into 5-μm sections which were stained with haematoxylin-eosin (H/E) and Masson’s trichrome (MT) for a histological assessment. The histological changes derived from three individual experiments were evaluated by using optical microscopy (Olympus BX51, Tokyo, Japan) in non-consecutive, randomly chosen ×200 histological fields. The digital photomicrographs were then processed with DP-72 (Olympus) [30].

### 2-DE and image analysis

Rat plasma protein (200 μg) was thawed and diluted in IPG sample buffer containing 7 M urea, 2 M thiourea, 4% CHAPS, 65 mM DTT, and 1% IPG buffer to a volume of 350 μl. Proteins were then loaded onto 18-cm IPG strips (pH 4∼7), IEF was automatically conducted with a total of 80 kVh after rehydration for 12 h at 30 V. Following IEF separation and equilibration, electrophoresis was carried out on 10% acrylamide gels at 40 mA. Proteins were visualized by silver staining and then scanned using an Imagescanner. Protein spots were quantified using the Prodigy SameSpots software (Nonlinear Dynamics, Newcastle, UK). The utility of constitutively expressed or trusted spots as a reference was expanded to assume the distribution of expression levels by its quantile within the gel. The raw intensity values were then normalized by their position in the cumulative distribution [31]. The protein spots with *p* values less than 0.05 as well as more than 1.5-fold alteration in the protein abundance between DMN2W and control were considered as “significant” changes. The proteome experiments were technically repeated from three independent experiments.

### In-gel digestion and MS analysis

Silver-stained spots were excised and in-gel-digested with trypsin according to previously described procedures [32]. Briefly, spots showing differential expression were manually excised and digested with trypsin (20 μg/mL) at 37°C overnight. After digestion, tryptic peptides were acidified with 0.5% TFA and loaded onto an MTP AnchorChip™ 600/384 TF (Bruker-Daltonik, Bremen, Germany). The MS analysis was performed on an Ultraflex™ MALDI-TOF mass spectrometer (Bruker-Daltonik). Monoisotopic peptide masses were assigned and used for Swiss-Prot primary sequence database searches with the BioTools 3.2 software (Bruker-Daltonik) and the Mascot search engine (http://www.matrixscience.com) (Matrix Science, London, UK). Search parameters were set as follows: a maximum allowed peptide mass error of 50 ppm and consideration of 1 incomplete cleavage per peptide. For MS/MS, the 3 most intense precursor ions with a signal/noise ratio of > 25 were selected after exclusion of the common background signal. The MS/MS mode was operated at 1 keV, and products of metastable decomposition at elevated laser power were detected. PMF data were acquired with close internal calibration and MS/MS data using the default instrument calibration.

### Biological network analysis using MetaCore™

MetaCore™ software (vers. 5.2 build 17389, GeneGo, St. Joseph, MI, USA) was performed to reveal the ontological classes and associated pathways which were represented among the proteins identified by the 2-DE and peptide mass fingerprint. Based on gene ontological categorization, two algorithms for the network analysis were applied: (i) an analysis network algorithm to deduce scoring processes regulated by differentially expressed proteins, and (ii) the shortest path algorithm for building a network consisting of the smallest possible number of direct interactions between different proteins. The statistical relevance of the ontological matches was calculated as the *p* value, which is the probability of a match occurring by chance, given the size of the database. The *p*-value was calculated using the following formula:

$$P-\mathrm{value}=\left[R!n!\left(Ν-R\right)!\left(Ν-n\right)!\right]/N!\sum_{i=\max\left(r,R+n-N\right)}^{\min\left(n,R\right)}1/\left[i!\left(R-i\right)!\left(N-R-n+i\right)!\right];$$

N is the total number of nodes in the MetaCore™ database; R is the number of network objects corresponding to genes and proteins in the list; n is the total number of nodes in each small network generated from the list; and r is the number of nodes with data in each small network [32].

### Rat cytokine protein array

The spectrum of cytokines produced by apparently control and DMN2W subjects was tested using an antibody-based protein microarray (RayBio™ Rat Cytokine Ab Array II, RayBiotech Inc., Norcross, GA) designed to detect 34 growth factors, cytokines of chemokines. Experiments were conducted as recommended by the manufacturer. GeneTools Image Software was applied to perform densiometric analysis. Data were normalized to a positive control spot on each array.

### Western blot analysis

Protein samples (30 μg) were separated with 10% SDS-PAGE and transferred to a PVDF membrane. Western blot analysis was performed using Haptoglobin (DAKO), ApoA4 (Bioss), ApoE (ABBIOTEC), ApoA1 and Albumin (Santa Cruz) overnight at 4°C. Blots were washed and incubated with HRP-labeled secondary antibody. Enhanced chemiluminescence was used for signal detection [29]. The Western blot experiments were repeated in triplicate.

### Quantification of targeted proteins in human plasma samples

To explore the clinicopathological significance of ApoA4, ApoA1 and Haptoglobin expression, we evaluated protein levels in human plasma samples derived from 22 healthy control and 33 liver fibrosis patients with fibrosis score less than stage 2. The information of clinical sample was shown in the Table 2. We have applied a separate cohort of 55 subjects (22 controls and 33 liver fibrosis patients) Under approval of Institutional Review Board, Chang Gung Memorial Hospital, Taiwan, plasma samples were retrospectively retrieved from the serum bank, Liver Research Center, Chang Gung Memorial Hospital for a study attempting to correlated biochemistry, tissue histology, and harmonic microscopy characteristics for liver fibrosis in hepatitis B patients. All patients included had previously received liver biopsy for evaluation of hepatitis activities and liver fibrosis [33]. The loading amount of protein was determined and normalized by using the Bradford Protein Assay Kit (AMRESCO). Western blotting assays were applied and quantified using GeneTools Image Software. The standard curve (R^2^ = 0.96) has been shown to ensure the accuracy for Western blotting assays as indicated in the Supplementary Figure 1. All experiments were technically repeated more than twice.

**Table 2:** Demographic data of patients with fibrosis and the control group

	Patients	Control	*P*
Age	40±2.5	37±3.2	
Sex	24 male, 9 female	14 male, 8 female	
AST (U/mL)	86.4±18.2	20.1±0.8	<0.001
ALT (U/mL)	160.2±36.4	12.3±1.3	<0.001
AFP (ng/mL)	5.71±1.55	2.85±1.63	
FibroScore	≤ 2	0	
FibroIndex	1.50±0.12	0.78±0.58	

### Hepatic stellate cell culture

HSC-T6 cell was a kind gift of Dr. Scott L. Friedman (Mount Sinai School of Medicine, New York, NY). The HSC-T6 cells were maintained in Waymouth medium containing 10% FBS at 37°C in a humidified atmosphere of 5% CO_2_. 1×10^6^ cells were seeded in petridish for 24 hours (h) and made quiescent by incubating in medium containing 0.2% FBS overnight. After treating with TGF-β1 (5 ng/mL; ProSpec-Tany TechnoGene, Israel) and ApoA4 (1 μg/mL; ALPHA DIAGNOSTIC, San Antonio, USA) for 24hr, the lysate was centrifuged at 10,000×g at 4°C for 20 min and the supernatant (50 μg of protein) was subjected to 12% SDS-PAGE. Primary antibodies, including ɑ-SMA, c-Myc, COL1A1 and β-actin (Santa Cruz) were used to evaluate the individual protein expression levels [29].

### Statistical analysis

All values of data derived from experiments were presented as the mean±SD. Statistical analysis of the mean values was carried out with the one way ANOVA. Differences were considered significant at *p<0.05. Statistical analysis using logistic regression and ROC curve was used as a measurement of discrimination between each parameter: ApoA1, ApoA4, haptoglobin (HPG) and the combination of parameters (ApoA4+HPG, ApoA1+ApoA4+HPG). It was estimated by using the nonparametric method by DeLong et al [34]. The analyses were performed using SPSS Version 21.0 for Windows (IBM, Armonk, NY, USA).

## SUPPLEMENTARY MATERIALS FIGURES



## Abbreviations

DMN: dimethylnitrosamine
ALT: alanine aminotransferase
AST: aspartate aminotransferase
ECM: extracellular matrix
TAA: thioacetamide
MCP-1: monocyte chemoattractant protein
IL-6: interleukin 6
TGF-β1: transforming growth factor beta 1
HSC: hepatic stellate cell
ROS: reactive oxygen species
TIMP: tissue inhibitors of metalloproteinase
α-SMA: alpha smooth muscle actin
COL1A1: alpha-1 type I collagen
Apo: apolipoprotein
H/E: haematoxylin-eosin
MT: Masson’s trichrome
IEF: isoelectric focusing
IPG: immobilized pH gradient
DTT: dithiothreitol
MS: mass spectrometry
CHAPS: 3-[(3-cholamidopropyl)dimethylammonio]-1-propanesulfonate
FBS: fetal bovine serum
CINC-1: cytokine-induced neutrophil chemoattractant-1
TTHY: transthyretin
VEGF: vascular endothelial growth factor
NF-κB: nuclear factor kappa-light-chain-enhancer of activated B cells
AP-1: activator protein 1
PPAR: peroxisome proliferator-activated receptors
ROC: receiver operating characteristic
AUC: area under the curve
HPT: haptoglobin
2-DE: two-dimensional electrophoresis
