# Supplementary material for: Proteome-based identification of apolipoprotein A-IV as an early diagnostic biomarker in liver fibrosis
Source: Oncotarget. 2017 Oct 6;8(51):88951–64. doi: 10.18632/oncotarget.21627 (PMC5687660; doi:10.18632/oncotarget.21627)
Supplement: Supplementary file 1 [file oncotarget-08-88951-s001.pdf]

Proteome-based identification of apolipoprotein A-IV as an early diagnostic biomarker in liver fibrosis

SUPPLEMENTARY MATERIALS

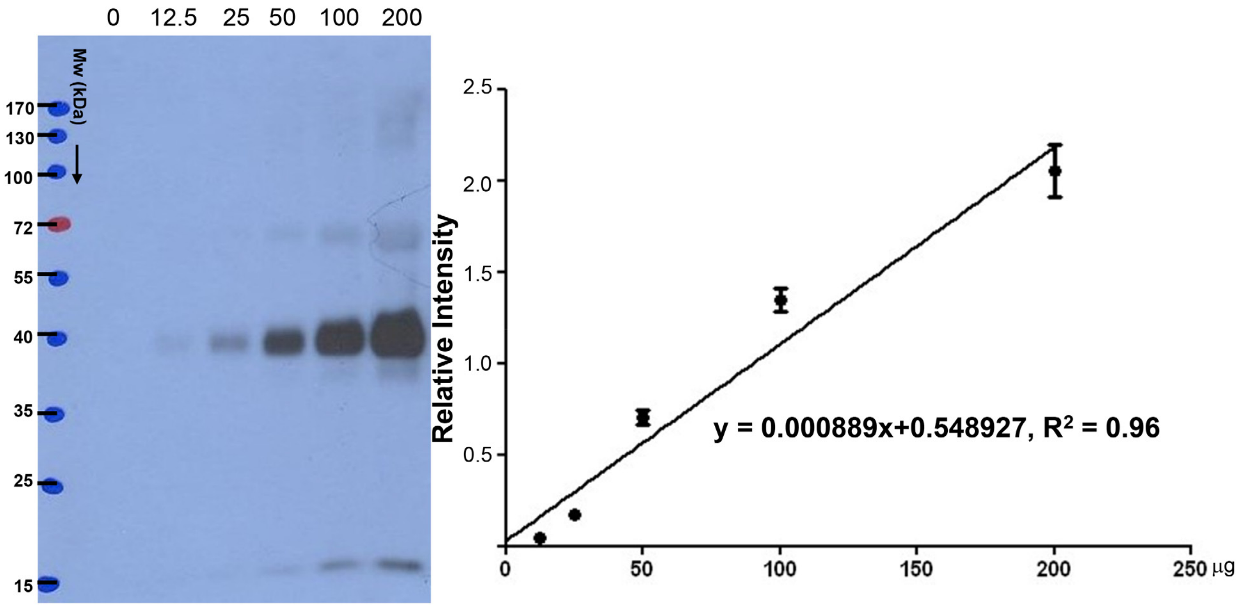

Supplementary Figure 1: Calibration curve indicating the average absorbance values in Western blotting experiments. The average blank value for 0 µg of protein is subtracted from each point.

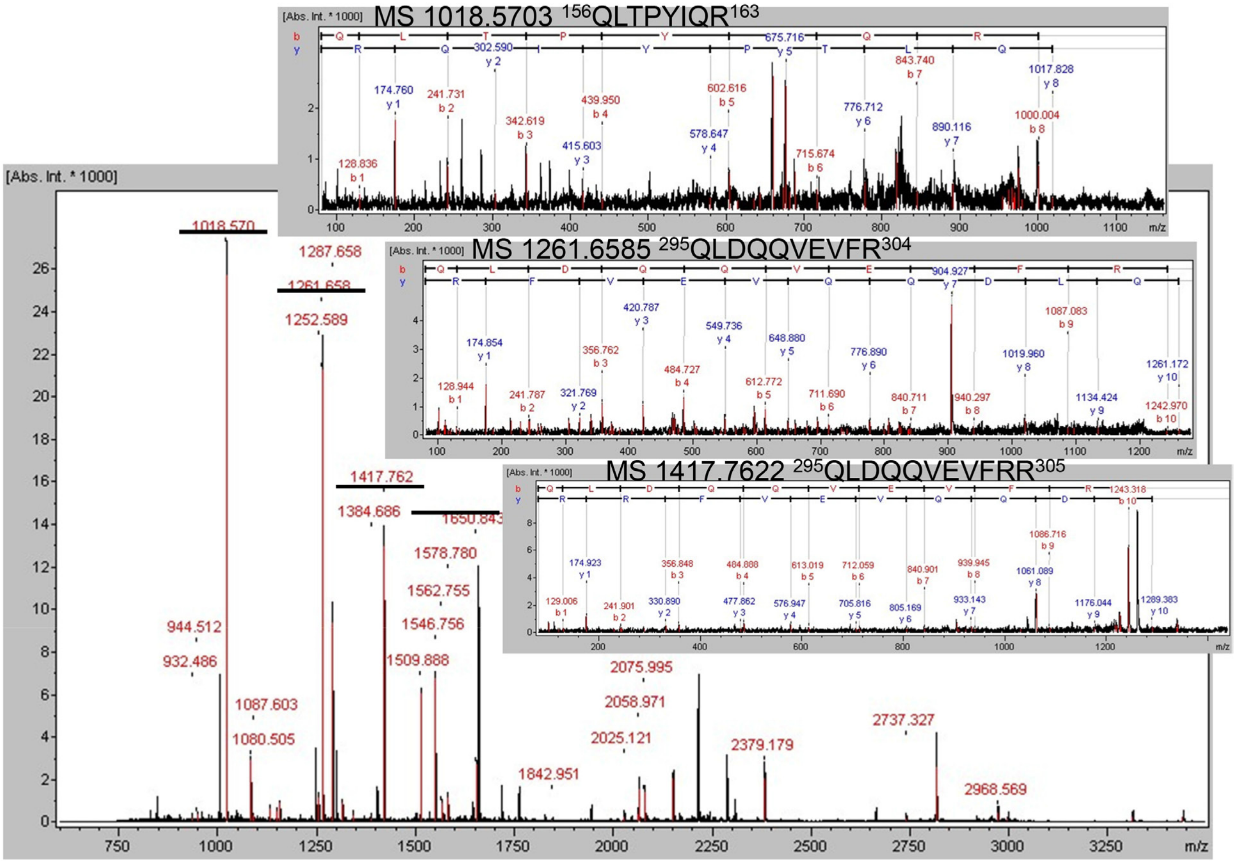

Supplementary Figure 2: MALDI-MS/MS spectra of ApoA4. MS spectrum of the ApoA4 protein was produced in the reflector mode for MALDI-MS. The parent ions m/z 1018.5703, 1261.6585, 1417.7622 were selected for further analysis by an Ultraflex™ MS/MS operated in the LIFT mode using FlexControl™ software. The amino acid sequences were unambiguously assigned to the rat ApoA4 protein. A sequence was confirmed from the labeled b- and y-ions in the spectrum.
